# Bleomycin Electrosclerotherapy for Peripheral Low-Flow Venous and Lymphatic Malformations in Children: A Monocentric Case Series

**DOI:** 10.3390/children12091167

**Published:** 2025-09-01

**Authors:** Edoardo Guida, Alessandro Boscarelli, Zeljko Zovko, Matea Peric-Anicic, Marianna Iaquinto, Maria-Grazia Scarpa, Sonia Maita, Damiana Olenik, Daniela Codrich, Jürgen Schleef

**Affiliations:** 1 Department of Pediatric Surgery and Urology, Institute for Maternal and Child Health—IRCCS “Burlo Garofolo”, 34137 Trieste, Italy; edoardo.guida@burlo.trieste.it (E.G.); marianna.iaquinto@burlo.trieste.it (M.I.); mariagrazia.scarpa@burlo.trieste.it (M.-G.S.); sonia.maita@burlo.trieste.it (S.M.); damiana.olenik@burlo.trieste.it (D.O.); daniela.codrich@burlo.trieste.it (D.C.); jurgen.schleef@burlo.trieste.it (J.S.); 2 Department of Surgery, University Hospital of Mostar, 88000 Mostar, Bosnia and Herzegovina; zeljko.zovko@mef.sum.ba (Z.Z.); matea.peric.anicic@mef.sum.ba (M.P.-A.)

**Keywords:** bleomycin, children, vascular malformations, electrochemotherapy, treatment

## Abstract

**Highlights:**

**What are the main findings?**

- Bleomycin electrosclerotherapy appears to be a promising option for treating peripheral low-flow vascular malformations with no potentially severe post-procedural complications.
- In certain cases, a single attempt could not be sufficient to achieve a complete recovery. However, previous treatments and/or pharmacological therapies do not appear to be a contraindication for bleomycin electrosclerotherapy.

**What is the implication of the main finding?**

- Bleomycin electrosclerotherapy is a feasible option for treating peripheral low-flow venous and lymphatic malformations with positive results regarding aesthetic aspects.
- Bleomycin electrosclerotherapy is a safe option for treating low-flow venous and lymphatic malformations in children.

**Abstract:**

**Background:** Vascular malformations are relatively common in children. Current therapeutic strategies include observation, medical therapy, sclerotherapy or embolization, laser therapy, cryoablation, and surgery, depending on the type and anatomical location of the malformation. Surgery is commonly limited to small and/or circumscribed lesions, to debulking in case of large volumes, or in drug-resistant cases. Sclerotherapy is a minimally invasive treatment generally used to treat dysplastic vasculature and to significantly improve patients’ symptoms. Herein, we describe our preliminary experience with bleomycin electrosclerotherapy (BEST) in the treatment of peripheral low-flow venous and lymphatic malformations in the pediatric population. **Methods:** We prospectively collected and analyzed data from patients who underwent BEST for peripheral low-flow vascular malformations (venous and lymphatic) and were treated at our institution from May 2022 onward. **Results:** Twelve patients (4 boys and 8 girls) with peripheral low-flow vascular malformations who underwent BEST were enrolled in this preliminary study. The median patient age at the first procedure was 81 months (IQR = 46–128). The most frequent anomaly was peripheral low-flow venous malformation. No relevant postoperative complications were encountered in any of the patients. All patients underwent a clinical evaluation of the malformation 1 month after the procedure. A clinical and ultrasonographic evaluation of the malformation was performed 2 months after the procedure to determine whether to repeat BEST. In cases of clinical resolution, a second ultrasonographic evaluation was performed 6 months after the procedure. **Conclusions:** BEST appears to be a promising and safe option for treating peripheral low-flow vascular malformations in children. Further studies with a greater number of patients and longer follow-up periods are needed to confirm our preliminary experience.

## 1. Introduction

Vascular anomalies are divided into two main categories according to the latest classification developed by the International Society for the Study of Vascular Anomalies (ISSVA): vascular tumors (benign, local aggressive/borderline, and malignant) and vascular malformations (simple, combined, of major named vessels, and associated with other anomalies). In particular, simple vascular malformations are subdivided into low-flow (capillary, lymphatic, and venous) and fast-flow (arteriovenous malformations and fistulae) lesions [1].

Vascular malformations are relatively common in children. In particular, low-flow venous malformations are encountered in approximately 1 per 1000 births. Clinical and sonographic evaluations are considered the gold standard approaches for diagnosing such anomalies; however, magnetic resonance imaging (MRI) studies are crucial for characterizing these anomalies to optimize therapy planning [2,3].

According to ISSVA classification system, simple venous malformations are divided into common venous malformations, familial venous malformation cutaneomucosal (VMCM), blue rubber bleb nevus syndrome venous malformation (BRBNS), glomuvenous malformation (GVM), and cerebral cavernous malformations (CCM). Venous malformations are most often sporadic, but VMCMs and GVMs are inherited in an autosomal dominant fashion. Particularly, the genetic basis for VMCMs and 40% of sporadic venous malformation is attributable to a mutation in the TEK gene (chromosome 9p), which binds angiopoietins and regulates angiogenesis, cell proliferation, migration, adhesion, and vascular quiescence via the TIE2-PI3K-AKT-mTOR pathway leading to disorganization of periendothelial cells and aberrant angiogenesis. Whereas, GVMs are caused by mutations in the glomulin gene, which is poorly understood [4].

Current therapeutic strategies include observation, medical therapy (e.g., compression therapy or mTOR inhibitors), sclerotherapy or embolization (e.g., alcohols, detergents, antitumor agents, doxycycline), laser therapy, cryoablation, and surgery, depending on the type and anatomical location of the malformation. Surgery is commonly limited to small and/or circumscribed lesions, to debulking in case of large volumes, or in drug-resistant cases. Sclerotherapy is a minimally invasive treatment generally used to treat dysplastic vasculature and to significantly improve patients’ symptoms. Interestingly, in a recent retrospective multicenter cohort study, bleomycin electrosclerotherapy (BEST) was confirmed to be a valuable option, yielding encouraging outcomes for treating low-flow venous and lymphatic malformations in children [4,5].

Herein, we describe our preliminary experience with BEST in treating peripheral low-flow vascular malformations in the pediatric population.

## 2. Materials and Methods

We prospectively collected and analyzed data from patients who underwent BEST for peripheral low-flow venous and lymphatic malformations and were treated at our institution from May 2022 onward.

Preoperative diagnostics were performed with Doppler ultrasonography (US) for superficial and small malformations using probes ranging from 10 to 15 MHz. According to our radiologists’ indications, an MRI study with a contrast agent was also performed for patients with large and deep malformations for a better characterization. We examined the following parameters: demographic data, vascular malformation characteristics, surgical details, number of procedures, outcomes, and complications.

Owing to the small number of patients, both normally and nonnormally distributed continuous variables are expressed as medians (interquartile ranges [IQRs]). Moreover, categorical variables are expressed as numbers and percentages.

### Surgical Technique

**Figure 1:**
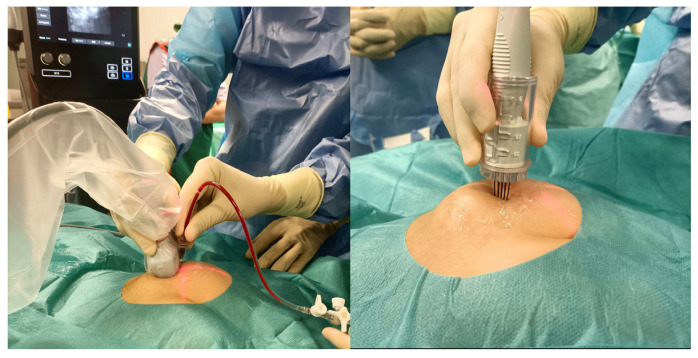
Intraoperative view of the intralesional injection of a solution of bleomycin and contrast agent under ultrasonographic guidance (**left**), follows by the application of electrical pulses generated by the electroporation device (**right**).

**Figure 2:**
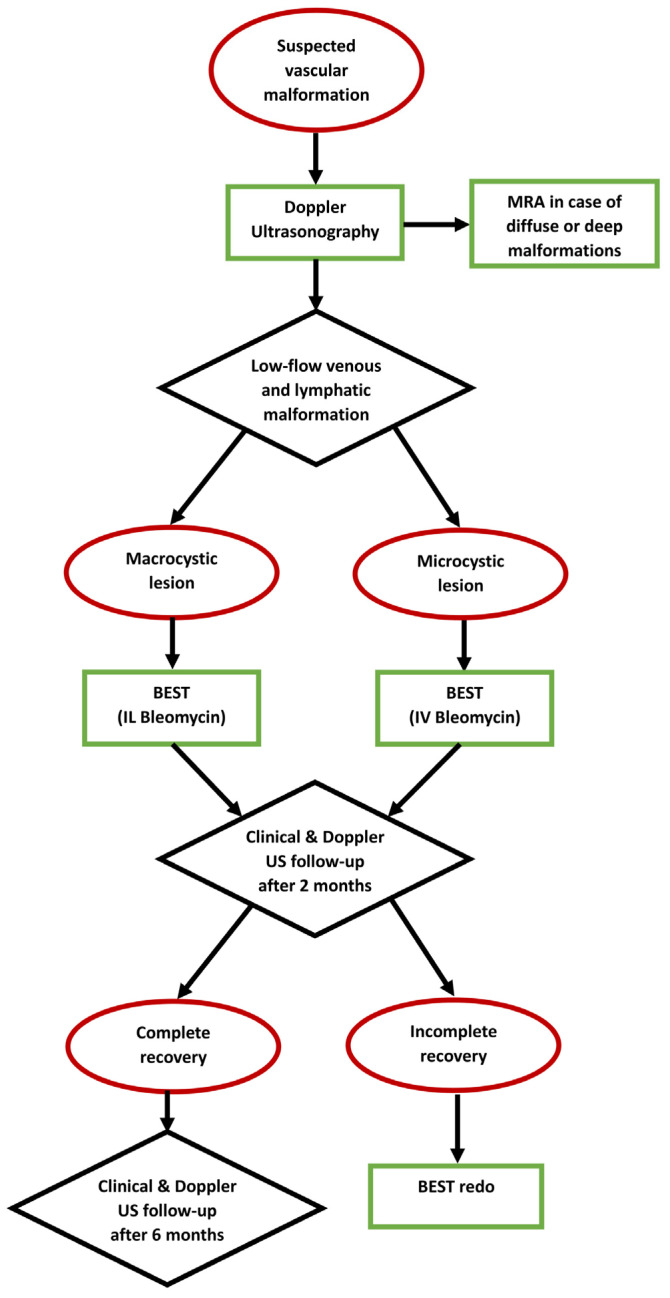
Diagnostic and management (with BEST) algorithm for pediatric patients with low-flow vascular malformations at our institution. (MRA = Magnetic Resonance Angiogragry; BEST = Bleomycin electrosclerotherapy; IL = Intralesional; IV = Intravenous; US = Ultrasonography).

The treatment was performed in a sterile operating environment under sedation or general anesthesia, depending on the patient’s age and vascular malformation site. In the case of macrocystic lesions, intralesional injection of a solution of bleomycin (with a bleomycin concentration of 1 mg/1 mL) and contrast agent (iopamidol) (with 1 mL of bleomycin diluted with 4 mL of iopamidol for a total of 20 mL of solution including a total of 5 mL of bleomycin) into the venous or lymphatic malformation was executed under ultrasonography/fluoroscopy guidance to verify the correct needle position and to study the anatomy of the vascular malformation. Bleomycin was injected at a dose of 0.5 mg/kg bleomycin, followed by the application of one or more electrical pulses generated by an electroporation device (IGEA S.p.A.) through electrodes with a specific design and adjustable length according to the lesion location and size (inserted into the lesion at a maximum depth of 4 cm) (Figure 1). In the case of microcystic lesions, endovenous infusion of pure bleomycin at a dose of 0.3 mg/kg bleomycin (with a bleomycin concentration of 1 mg/mL) was performed. Ten minutes after the endovenous infusion, one or more electrical pulses were applied. Figure 2 shows the diagnostic and therapeutic (with BEST) algorithm for low-flow vascular malformations in our tertiary-level pediatric surgical department.

## 3. Results

Twelve patients (4 boys and 8 girls) with peripheral low-flow vascular malformations were prospectively enrolled in this preliminary study. The median patient age at the first procedure was 81 months (IQR = 46–128). Notably, the youngest patient was under 1 year of age, whereas the oldest was aged 15 years.

**Figure 3:**
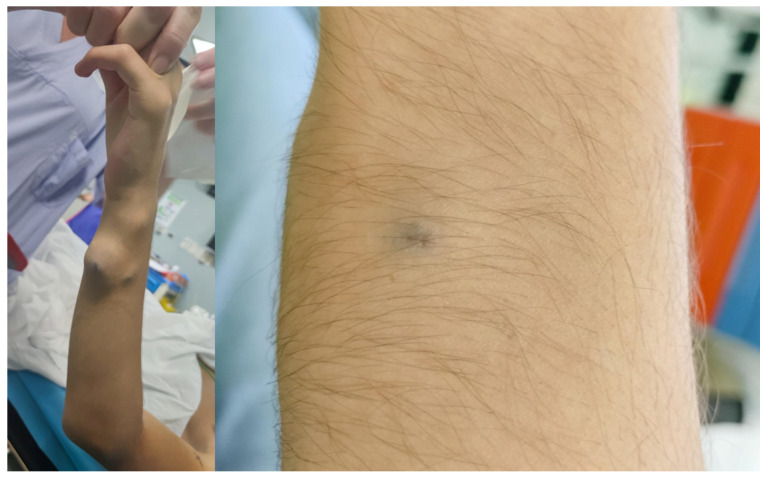
Macroscopic venous malformation of the right forearm treated with intralesional injection of bleomycin followed by electrosclerotherapy (**left**). Close-up view demonstrating a complete disappearance of the lesion observed at the 2-month follow-up (**right**).

**Figure 4:**
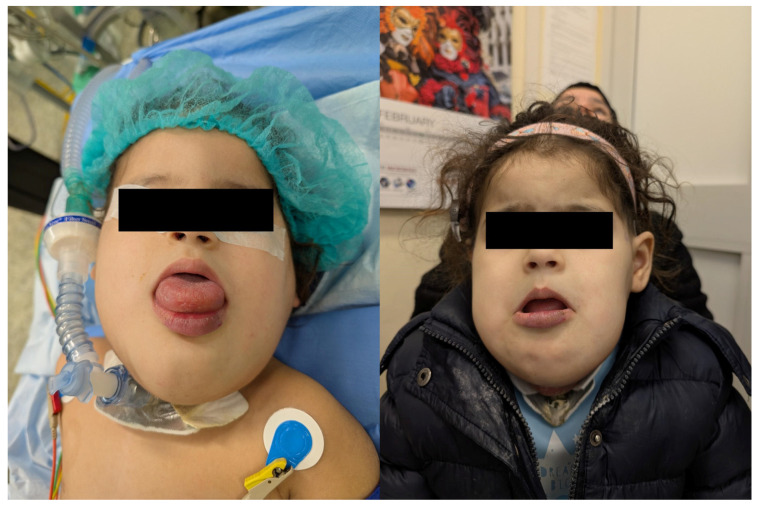
Microcystic lymphatic malformation of the tongue treated with endovenous injection of bleomycin followed by electrosclerotherapy 10 min after the injection (**left**). At the 1-month follow-up, the tongue size was significantly reduced, and the patient could close her mouth (**right**).

The most frequent anomaly was peripheral low-flow venous malformation (LFVM), presenting in 9 patients (75%). While, two patients (17%) presented with a lymphatic malformation and one patient (8%) with a mixed malformation. The anomalies were localized in the neck in 3 patients (25%) with one of them presenting also an involvement of the tongue, in the upper limbs in 2 (17%) patients (Figure 3), in the lower limbs in 2 patients (17%), in the subcutaneous tissue of the plantar face of the right foot in 2 patients (17%), in the right knee, subcutaneous tissue of the right buttock, and in the lumbosacral region in one (8%) patient, respectively. Interestingly, two out of 12 patients (17%) presented with a large macrocystic lymphatic malformation on the left side of the neck and with diffuse mixed macrocystic and microcystic lymphatic malformations of the entire neck involving the tongue, respectively (Figure 4). Remarkably, only two patients (17%) experienced a self-limiting skin dyschromia and a hematoma of the tongue in the postoperative period followed by a full recovery in both cases.

Five out of twelve patients (42%) were discharged home the same day of the procedure. All patients underwent a clinical evaluation of the malformation 1 month after the procedure. A clinical and ultrasonographic evaluation of the malformation was performed 2 months after the procedure to determine whether to repeat BEST. In cases of clinical resolution, a second ultrasonographic evaluation was performed 6 months after the procedure. Notably, patients with venous malformations in the right lower limb and lumbosacral region required two procedures. In addition, two patients with large lymphatic malformations of the neck have required four procedures to date.

A minimum follow-up of 6 months was performed for 6 patients with a complete recovery in all cases, and a 5-month follow-up was performed for two patients after the last bleomycin injection, revealing a good reduction in the malformation volume and satisfying esthetic results. Four out of 12 patients are still being followed and treated at our institution to date.

Detailed data about patients enrolled in our prospective study to date are reported in Table 1.

## 4. Discussion

The term electrochemotherapy (ECT) was first coined in 1991 by a group of researchers at the biochemical laboratory of the Institute Gustave-Roussy in France. Their study described the effect of reversible electroporation of cell membranes obtained through local application of electric pulses combined with highly cytotoxic drugs with limited or no transport across the plasma membrane. Accordingly, a hydrophilic antibiotic with endonuclease activity, such as bleomycin, is considered an optimal choice [6,7,8]. Given the antitumor effectiveness of BEST demonstrated in preclinical studies, this approach was consequently included in the European guidelines for treating melanoma, colorectal cancer metastases and skin tumors [8]. The use of BEST to treat venous malformations in the pediatric population has also been initiated, with encouraging results. Hence, the International Network for Sharing Practices on Electrochemotherapy (InspECT) has recently created a dedicated working group to standardize the procedure and promote clinical trials. However, studies on BEST for treating peripheral low-flow vascular malformations in children are rare, and few have reported results in both pediatric and adult populations [5,9,10]. It is well-known that ECT enhances effects of various drugs while enabling the use of lower dosages and consequently minimizing the adverse effects. Further, it can also be used for patients who before received alternative therapies and/or chemotherapy in drug-resistant cases. Nonetheless, patients receiving BEST must be followed-up carefully after treatment. In fact, pulmonary toxicity is reported to be a potential long-term side effect in the use of bleomycin and an area of great concern. Thus, BEST should be used with extreme caution in patients with impairment of renal or pulmonary functions [11,12].

This preliminary study focused exclusively on pediatric patients investigating BEST, which is already considered standard of care in several specialized vascular anomaly centers, as a treatment opportunity for peripheral low-flow vascular malformations (venous and lymphatic). First, although a new technology characterized by the use of probes ranging from 30 to 100 MHz (ultra-high frequency ultrasonics, UHFUS) demonstrating accuracy comparable to a biopsy was introduced in the clinical setting in the early 2000 s [13,14], our radiologists continue to obtain preoperative imaging studies using ultrasound with probes in a range of 10 to 15 MHz. In fact, UHFUS provides a higher spatial resolution at the expense of a lower penetration depth and we believe that classical ultrasound is sufficient to study most superficial lesions while for deeper ones an MRI study is still necessary. Second, based on our preliminary experience, we learned that in lesions with microcystic aspects where intralesional bleomycin injection is challenging due to the small cyst size intravenous bleomycin injection with electroporation 10 min after injection initiation should be considered and could improve results. Third, we noted that in certain cases a single attempt could not be sufficient to achieve a complete recovery and that previous treatments and/or pharmacological therapies do not appear to be a contraindication for Bleomycin electrosclerotherapy. In detail, two patients in our case series presented an isolated lymphatic malformation. One of them had previously undergone administration of intralesional OK-432 (Picibanil) while the other is still on oral therapy with Sirolimus. More specifically, both patients needed several attempts, and we switched from an intralesional approach to an intravenous approach based on modification of cysts dimensions. Both of them are still being treated at our institution. Overall, a positive result was obtained regarding esthetic aspects, with no potentially severe post-procedural complications. In particular, consistent with the findings of a recent study by Schmidt and colleagues [5], only two patients in our case series experienced a post-treatment side effect that resolved spontaneously without any sequelae.

The main limitation of this study is the small sample size, which limits the generalizability of our findings. In particular, 4 out of 12 patients enrolled in the present study are still under therapy, and long-term results are currently unknown. Hence, long-term follow-up could provide additional and noteworthy information regarding patient outcomes.

## 5. Conclusions

BEST appears to be a promising and safe option for treating peripheral low-flow vascular malformations in children. To date, we have noticed an overall reduction in the dimensions of the malformations, although a few patients are still being followed up and may require multiple procedures. Further studies with a greater number of patients and longer follow-up periods are needed to confirm our preliminary experience.

## Figures and Tables

**Table 1:** Characteristics of patients followed for a low-flow venous or lymphatic malformation at our institution and treated with BEST (IL = intralesional; IV = intravenous; US = Ultrasound).

#	Gender	Age @ Surgery (Months)	Type	Localization	Previous Treatments	Administration	Total Attempts	Complications	Results
1	F	66	Venous	Right buttock	None	Intralesional	1	No	Recovery
2	M	57	Venous	Left lower limb	None	Intralesional	1	No	Recovery
3	M	187	Venous	Left upper limb	None	Intralesional	1	temporary skin dyschromia	Recovery
4	F	142	Venous	Right foot	None	Intralesional	1	No	Recovery
5	F	114	Venous	Right upper limb	None	Intralesional	1	No	Recovery
6	M	182	Venous	Right lower limb	None	Intralesional	2	No	small vascular loopholes at US
7	F	9	Lymphatic	Neck	IL Picibanil (OK-432)	Intralesional	4 (3 IL, 1 IV)	No	ongoing
8	F	35	Lymphatic	Neck and tongue	Sirolimus per os	Intralesional	4 (2 IL, 2 IV)	Hematoma of the tongue	ongoing
9	F	87	Venous	Right knee	None	Intralesional	1	No	small vascular loopholes at US
10	M	34	Venous	Right foot	None	Intravenous	1	No	ongoing
11	F	76	Venous	Lumbosacral	None	Intralesional	2	No	ongoing
12	F	86	Venous lymphatic	Neck	None	Intralesional	1	No	Recovery

## Data Availability

The data presented in this study are available on request from the corresponding author due to privacy, legal, and ethical reasons.

## Abbreviations

The following abbreviations are used in this manuscript:

BEST	Bleomycin electrosclerotherapy
MRI	Magnetic resonance imaging
VMCM	Venous malformation cutaneomucosal
GVM	Glomuvenous malformation
LFVM	Low-flow venous malformation
US	Ultrasonography
ECT	Electrochemotherapy
UHFUS	Ultra-high frequency ultrasonics
